# Draft Genome Sequence of the Chloroperoxidase-Producing Fungus *Caldariomyces fumago* Woronichin DSM1256

**DOI:** 10.1128/genomeA.00774-16

**Published:** 2016-08-04

**Authors:** Harald Kellner, Marek Jan Pecyna, Markus Buchhaupt, René Ullrich, Martin Hofrichter

**Affiliations:** a Department of Bio- and Environmental Sciences, International Institute Zittau, Technische Universität Dresden, Zittau, Germany; b DECHEMA Research Institute, Biochemical Engineering, Frankfurt am Main, Germany

## Abstract

We report here the draft genome sequence of the chloroperoxidase (EC 1.11.1.10)-producing ascomycete *Caldariomyces fumago*. Its genome was assembled into 511 contigs with a total size of 25 Mb. The G+C content is 51.4%, and 9,806 putative protein-coding genes were predicted. Eight heme-thiolate peroxidase genes, including two chloroperoxidase genes, were found.

## GENOME ANNOUNCEMENT

The filamentous fungus *Caldariomyces fumago* (syn. *Leptoxyphium fumago*) is known for its ability to produce the biotechnologically relevant enzyme chloroperoxidase (CPO, EC 1.11.1.10), which was discovered in 1959 (1–4). The presence of this enzyme also triggered research on the fungus and its physiology. One halogen-containing metabolite in cultures of *C. fumago* was named caldariomycin, a cyclopentane derivative containing two chlorine atoms (5). Some other chlorinated metabolites from *C. fumago* were identified (6), and a dichlorinated diketopiperazine from this fungus was discovered during a screen for substances with a specific pharmacological effect (7). After the isolation of caldariomycin, the group of Lowell Hager was successful in demonstrating the ability of CPO to chlorinate potential precursors of caldariomycin (1–3, 8, 9), although the biosynthetic pathway of this compound has not been clarified to date. Until today, two CPO genes were elucidated (10), and genetic manipulation for this fungus was established (11). Nevertheless, for further genetic research and manipulations, a draft genome of this fungus will be the logical next step.

*C. fumago* DSM1256 (ribosomal cistron accession no. KX289331) was obtained from the Leibniz Institute German Collection of Microorganisms and Cell Cultures (DSMZ, Braunschweig, Germany). The strain was cultivated in 2% malt medium in agitated flasks at room temperature. Biomass was harvested and freeze-dried, and DNA was extracted using the DNeasy plant maxi kit (Qiagen, Hilden, Germany). Genomic DNA was enzymatically sheared, and a 200-bp fragment library was prepared using the Ion Xpress Plus fragment library kit (Thermo Fisher, Darmstadt, Germany). After emulsion-based PCR (emPCR; Ion PGM template OT2 200 kit), the Ion Torrent PGM was used for sequencing (Ion PGM sequencing 200 kit version 2, 318v2 Chip). Four million reads with a median size of 218 bp were generated.

The assembly of 3.7 million reads 80 to 300 bp in size was performed using MIRA4.0 (12) integrated in Geneious R8 (13). A second assembly step using the Geneious R8 assembler (setting, high sensitivity) was conducted to filter for duplicate contigs. Assembly quality was analyzed using CEGMA (14), and the best assembly showed 95% completeness of full-length conserved genes. Altogether, 511 contigs, with a size of 25 Mb and a coverage of 27×, were generated. Genome statistics were evaluated using QUAST (15), with an *N*_50_ of 186,141 and a G+C content of 51.44%. Augustus (16) was used to predict 9,806 protein-coding genes. Blast2GO (BioBam, Valencia, Spain) was used to annotate the proteins and to find heme-thiolate peroxidases/peroxygenases of interest. Altogether, eight heme-thiolate peroxidase genes, including the two chloroperoxidase genes, were found (accession numbers KX289323 to KX289330). Four of the eight heme-thiolate peroxidases belong to the short unspecific peroxygenases, and the remaining four belong to the long unspecific peroxygenases (17).

### Nucleotide sequence accession numbers.

This whole-genome shotgun project has been deposited at DDBJ/ENA/GenBank under the accession no. LSHF00000000. The version described in this paper is version LSHF01000000.
